# *DiGeorge syndrome critical region gene 2* (*DGCR2*), a schizophrenia risk gene, regulates dendritic spine development through cell adhesion

**DOI:** 10.1186/s13578-023-01081-9

**Published:** 2023-07-21

**Authors:** Dongyan Ren, Bin Luo, Peng Chen, Lulu Yu, Mingtao Xiong, Zhiqiang Fu, Tian Zhou, Wen-Bing Chen, Erkang Fei

**Affiliations:** 1 School of Life Sciences, Nanchang University, Nanchang, 330031 China; 2 Institute of Life Science, Nanchang University, Nanchang, 330031 China; 3 School of Basic Medical Sciences, Nanchang University, Nanchang, 330031 China

**Keywords:** DiGeorge syndrome critical region gene 2 (DGCR2), 22q11.2 deletion syndrome (22q11DS), Anxiety, Cell adhesion, Dendritic spine

## Abstract

**Background:**

Dendritic spines are the sites of excitatory synapses on pyramidal neurons, and their development is crucial for neural circuits and brain functions. The spine shape, size, or number alterations are associated with neurological disorders, including schizophrenia. *DiGeorge syndrome critical region gene 2* (*DGCR2*) is one of the deleted genes within the 22q11.2 deletion syndrome (22q11DS), which is a high risk for developing schizophrenia. DGCR2 expression was reduced in schizophrenics. However, the pathophysiological mechanism of DGCR2 in schizophrenia or 22q11DS is still unclear.

**Results:**

Here, we report that DGCR2 expression was increased during the neurodevelopmental period and enriched in the postsynaptic densities (PSDs). DGCR2-deficient hippocampal neurons formed fewer spines. In agreement, glutamatergic transmission and synaptic plasticity were decreased in the hippocampus of DGCR2-deficient mice. Further molecular studies showed that the extracellular domain (ECD) of DGCR2 is responsible for its transcellular interaction with cell adhesion molecule Neurexin1 (NRXN1) and spine development. Consequently, abnormal behaviors, like anxiety, were observed in DGCR2-deficient mice.

**Conclusions:**

These observations indicate that DGCR2 is a novel cell adhesion molecule required for spine development and synaptic plasticity, and its deficiency induces abnormal behaviors in mice. This study provides a potential pathophysiological mechanism of DGCR2 in 22q11DS and related mental disorders.

**Supplementary Information:**

The online version contains supplementary material available at 10.1186/s13578-023-01081-9.

## Introduction

Schizophrenia is a neurodevelopmental disorder characterized by marked disruption in perception, cognition, and motivation [1, 2]. These impairments may result from abnormal neuronal connectivity and plasticity [3]. Dendritic spines are the sites on the dendrites of pyramidal neurons where most glutamatergic excitatory synapses are located [4, 5]. Proper dendritic spine development in number and morphology is crucial for neural circuits and brain functions in adulthood. Dendritic spine shape, size, or number alterations are associated with neurodevelopmental disorders like mental retardation, autism spectrum disorders, and schizophrenia [5–10]. In schizophrenia, dendritic spine density is decreased in the cortex and hippocampus [8, 10, 11]. Thus, hypofunction of dendritic spine development may contribute to the pathogenesis of schizophrenia.

The 22q11.2 deletion syndrome (22q11DS), also known as velocardiofacial syndrome or DiGeorge syndrome, is a neurogenetic condition caused by a microdeletion in chromosome 22, with an incidence of 1 in 2,000–4,000 live births [12, 13]. 22q11DS is a high risk for schizophrenia. About 23 ~ 43% of patients with 22q11.2 deletion develop schizophrenia [14–16]. Nearly all orthologs of the deleted genes exist in the syntenic region of mouse chromosome 16 [17]. Deleting this region in mice results in schizophrenia-related abnormal behaviors, like impaired sensorimotor gating and working memory, which can be attributed to synaptic malfunctions [18–20]. *DiGeorge syndrome critical region gene 2* (*DGCR2*) is one of the deleted genes among the critical region and is also associated with schizophrenia. An Ashkenazi Jewish population study has found that several single nucleotide polymorphisms (SNPs) within *DGCR2* are associated with schizophrenia, and the risk allele of one SNP has a reduced expression level in the brain [21]. An exome sequencing study found that a *de novo* mutation in *DGCR2* is associated with schizophrenia [22]. These studies indicate *DGCR2* is a schizophrenia risk gene. *DGCR2* encodes a single transmembrane putative adhesion receptor protein [23]. Recently, it was found that DGCR2 regulates corticogenesis and cortical circuit development [24, 25]. However, little is known about the functions of DGCR2 in the synapse.

In the present study, we report that DGCR2 was a synaptic cell adhesion molecule localized to the postsynaptic densities (PSDs). Knocking down DGCR2 impaired spine development in hippocampal neurons; in agreement, *Dgcr2* mutant (*mt*) mice displayed fewer spines. Both glutamatergic transmission and synaptic plasticity were reduced in *mt* mice. Further molecular studies suggest that DGCR2 regulated spine development through cell adhesion. Consequently, abnormal behaviors, like anxiety, were observed in *mt* mice. Overall, our study provides a potential pathophysiological mechanism of DGCR2 in 22q11DS and related mental disorders.

## Results

### Localization of DGCR2 to the PSDs

To better understand the function of DGCR2, we examined its expression pattern in mice by western blotting (WB). As shown in Additional file 1: Figure S1A, DGCR2 was detected in the brain and several peripheral tissues, including the spinal cord, muscle, liver, and spleen. In the brain, DGCR2 was abundant in the cortex, hippocampus, cerebellum, olfactory bulb, hypothalamus, and striatum (Additional file 1: Figure S1B). During development, DGCR2 expression was increased in postnatal stage (Additional file 1: Figure S1C), a period of synapse development and maturation. To further characterize DGCR2 expression in the brain, we obtained *Dgcr2* mutant mice (*Dgcr2*-LacZ) from the European Conditional Mouse Mutagenesis Program (EUCOMM) [26]. In *Dgcr2*-LacZ mice, a cassette containing LacZ was inserted between exons 1 and 2 of the *Dgcr2* gene (Additional file 2: Figure S2A). Expression of LacZ is under the endogenous promoter of the *Dgcr2* gene and was expected to indicate the expression of *Dgcr2* faithfully. As shown in Additional file 2: Figure S2B, LacZ activity was mainly expressed in the hippocampus, cortex, thalamus, and hypothalamus. Because of the insertion of the LacZ cassette, the level of DGCR2 in *Dgcr2*-LacZ homozygous mutant was reduced by 30 ~ 40% (Additional file 2: Figure S2C-D). Unless otherwise indicated, homozygous LacZ/LacZ mice were referred to as *Dgcr2* mutant (*mt*) mice. Together, these results suggest that DGCR2 is expressed in various brain regions, and the expression is higher in the postnatal stage.

**Fig. 1:**
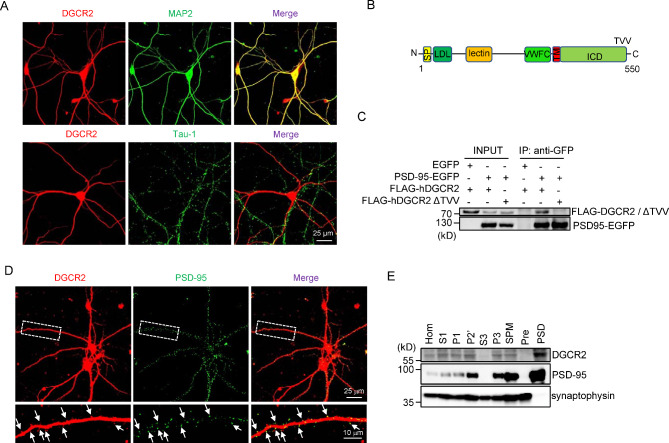
Localization of DGCR2 to the PSDs. **A** Colocalization of DGCR2 with MAP2. DIV19-20 hippocampal neurons were co-stained with anti-DGCR2 and anti-MAP2 (a dendrite marker) or anti-Tau-1 (an axon marker) antibody. Scale bar as indicated. **B** Schematic illustration of DGCR2 domain structure. SP, signal peptide; LDL, Low-Density Lipoprotein (LDL) receptor binding domain similarity; lectin, C-type lectin similarity; VWFC, von Willebrand factor (VWF) type C repeat; TM, transmembrane domain; ICD, intracellular domain; TVV, amino acids for threonine, valine, valine. **C** Binding of DGCR2 with PSD-95 in HEK 293T cells. Cells were co-transfected with PSD-95-EGFP or EGFP alone, and FLAG-hDGCR2 or FLAG-hDGCR2 ∆TVV. Lysates were immunoprecipitated with anti-GFP antibody and Protein A/G agarose, and resulting complexes were blotted with anti-GFP and anti-FLAG antibodies. Inputs were blotted for perspective proteins as controls. **D** Colocalization of DGCR2 with PSD-95 puncta. DIV19-20 hippocampal neurons were co-stained with anti-DGCR2 and anti-PSD-95 (a PSDs marker) antibodies. Scale bar as indicated. **E** Enriched expression of DGCR2 in the PSDs. Subcellular fractions of adult *wt* mouse brains were blotted with anti-DGCR2, anti-PSD-95 and anti-synaptophysin (a presynaptic marker). S1, supernatant 1; P1, pelleted nuclear fraction; P2’, washed crude synaptosomal fraction; S3, crude synaptic vesicle fraction; P3, lysed synaptosomal membrane fraction; SPM, synaptosomal membrane fraction; Pre, presynaptic fraction; PSD, postsynaptic density fraction

To observe the subcellular localization of DGCR2 in neurons, primary cultured hippocampal neurons were co-stained for DGCR2 and MAP2 (a dendrite marker) or Tau-1 (an axon marker). DGCR2 was mainly enriched in dendrites but not axons (Fig. 1A). DGCR2 is a single transmembrane protein with an amino (N)-terminal extracellular domain (ECD), a transmembrane domain and a carboxyl (C)-terminal intracellular domain (ICD) (Fig. 1B). The last three amino acid residues of the ICD (-TVV*) appeared to be a consensus motif (-T/SXV*, X could be any amino acid residue) that is critical for binding to PDZ domains [27]. To determine whether DGCR2 could interact with PDZ-containing proteins, human embryonic kidney (HEK) 293T cells were co-transfected with FLAG-tagged human DGCR2 (FLAG-hDGCR2) and EGFP-tagged PSD-95 (PSD-95-EGFP), a well-characterized synaptic PDZ protein [28]. Indeed, FLAG-hDGCR2 was co-immunoprecipitated with PSD-95-EGFP, indicating the interaction of these proteins (Fig. 1C). Deletion of the last three amino acid residues (∆TVV) prevented this interaction (Fig. 1C), demonstrating that the interaction is mediated by the PDZ-interacting motif. Moreover, DGCR2 co-localized with PSD-95 puncta in primary hippocampal neurons (Fig. 1D). Finally, via PSD fractionation assay, we found DGCR2 concentrated in the PSDs, but not in the presynaptic membrane fraction (Fig. 1E). These results suggest that DGCR2 is a synaptic protein enriched in the PSDs.

### Reduced dendritic spine density in neurons lacking DGCR2

**Fig. 2:**
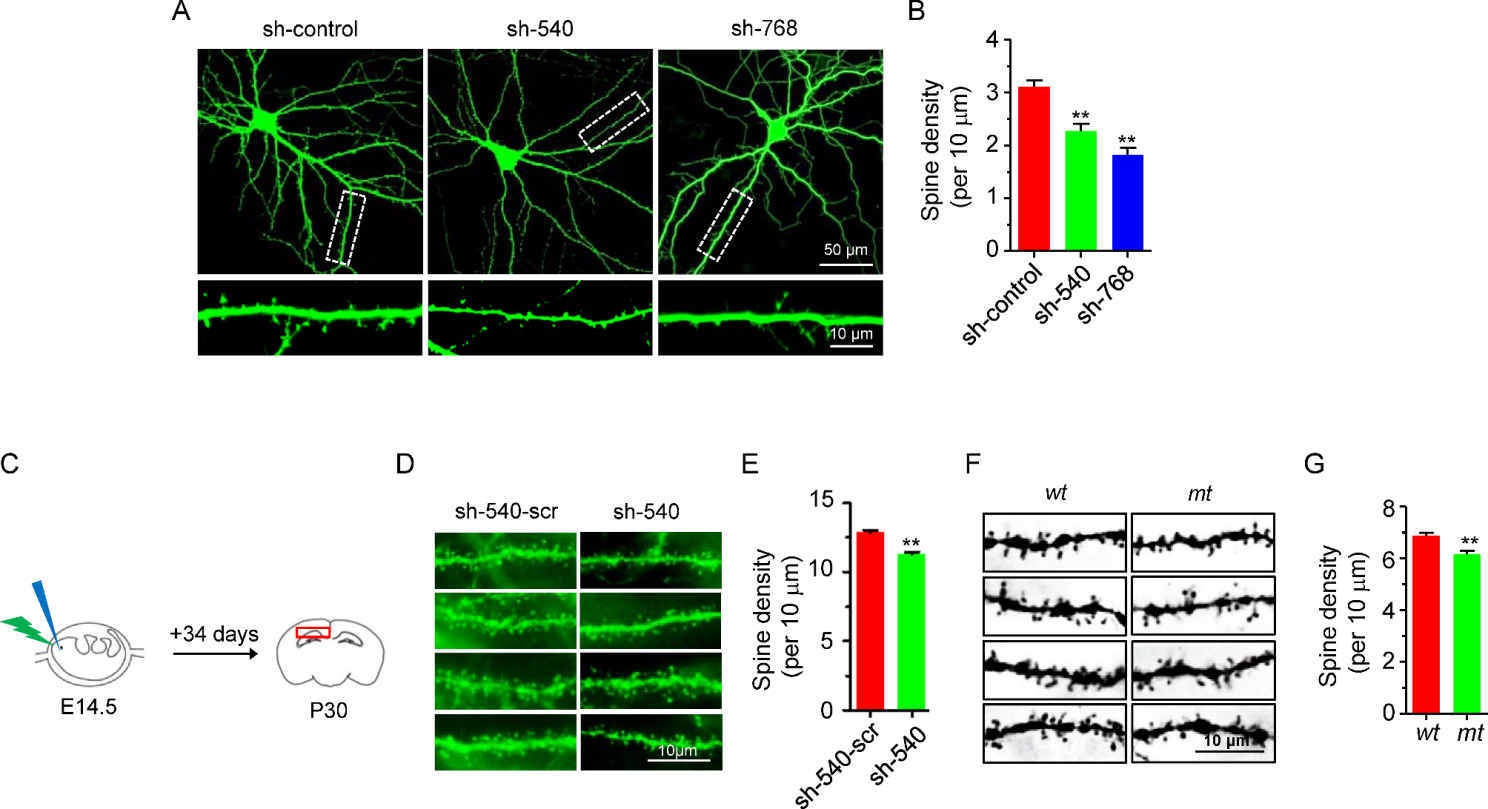
Reduced dendritic spine density in neurons lacking DGCR2. **A-B** Reduced spine density by DGCR2 knockdown in primary cultured hippocampal neurons. Representative images of neuronal morphology (upper panel) and dendritic spines (lower panel) of cultured hippocampal neurons (**A**). Neurons were isolated from rat E18.5 embryos, transfected at DIV9 with indicated constructs, and fixed for staining at DIV16. Sh-control, empty pSUPER-vector. Scale bars as indicated. Spine density (per 10 μm) quantitative analysis of data in A (**B**). n = 15 neurons for each condition. ** p < 0.005, One-way ANOVA. **C-D** Reduced spine density by DGCR2 knockdown in the hippocampus in vivo. Representative images of dendritic spines of electroporated hippocampal CA1 neurons (**C**). *In-utero* electroporation of DGCR2 shRNA (sh-540) and its control scramble shRNA (sh-540-scr) to the hippocampi of E14.5 or E15.5 embryos. At P30 after birth, sections were stained with anti-GFP antibody and subjected to spine analysis. Scale bars as indicated. Spine density quantitative analysis of data in C (**D**). n = 30 neurons for each condition. ** p < 0.005, Student’s *t* test. **E-F** Reduced spine density in *Dgcr2 mt* mice. Representative spine images from Golgi staining (**E**). Dendrite segments were chosen from hippocampal CA1 pyramidal neurons. Scale bars as indicated. Spine density quantitative analysis of data in E (**F**). n = 35 neurons for *wt* and n = 40 neurons for *mt*. ** p < 0.005, Student’s *t* test

Dendritic spine density is reduced in schizophrenics. Considering that *DGCR2* expression is decreased in schizophrenics, we determined whether its deficiency alters spine formation. To this end, we designed two small hairpin RNAs (shRNAs: sh-540 and sh-768) to knock down DGCR2 in hippocampal neurons. As shown in Additional file 3: Figure S3A-B, sh-540 and sh-768 were able to reduce the level of endogenous DGCR2 by ~ 40%. Noticeably, neurons transfected with shRNA displayed fewer spines than those transfected with sh-control (Fig. 2A-B). To determine whether in vivo spine formation requires DGCR2, we introduced sh-540 to the embryonic hippocampus by *in-utero* electroporation. As shown in Fig. 2C-E, sh-540, but not its scramble shRNA (sh-540-scr), reduced the spine density of CA1 pyramidal neurons. In agreement with in vitro and in vivo knockdown studies, the spine density, revealed by Golgi staining, was reduced in the hippocampus of *Dgcr2 mt* mice, compared with control wild type (*wt*) mice (Fig. 2F-G). Together, these results suggest that DGCR2 is critical for dendritic spine formation.

### Impaired glutamatergic transmission and synaptic plasticity in ***Dgcr2*** mutant mice

**Fig. 3:**
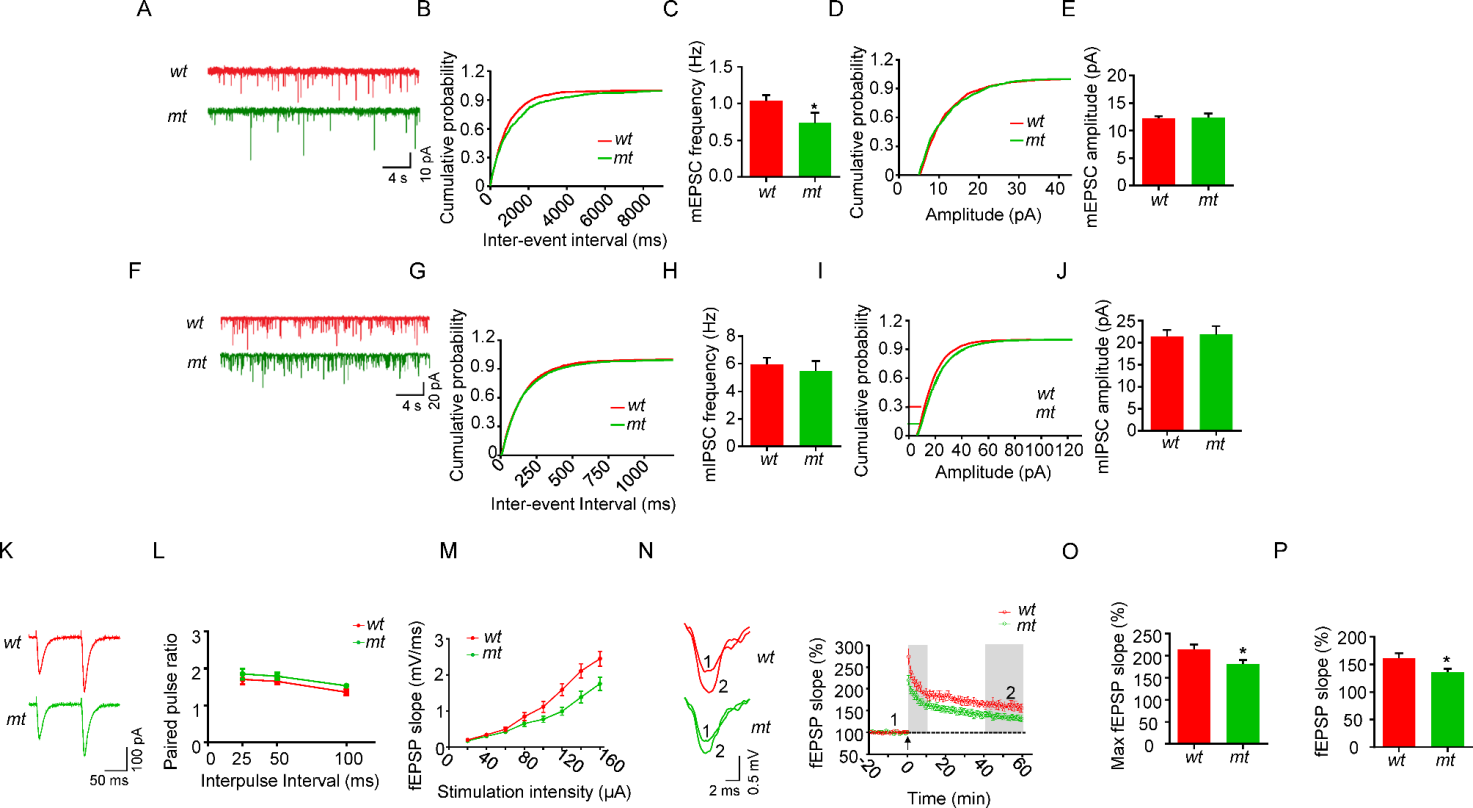
Impaired glutamatergic transmission and synaptic plasticity in *Dgcr2 mt* mice. **A** Representative traces of mEPSCs in CA1 pyramidal neurons from *wt* and *mt* mice. Scale bars represent 4 s, 10 pA. **B-E** Cumulative probability plots of mEPSC inter-event intervals and histograms of mEPSC frequency (**B** and **C**) and amplitude (**D** and **E**). n = 18 neurons from 4 *wt* mice, n = 13 neurons from 4 *mt* mice. **F** Representative traces of mIPSCs in CA1 pyramidal neurons from *wt* and *mt* mice. Scale bars represent 4 s, 20 pA. **G-J** Cumulative probability plots of mIPSC inter-event intervals and histograms of mIPSC frequency (**G** and **H**) and amplitude (**I** and **J**). n = 15 neurons from 4 *wt* mice, n = 12 neurons from 4 *mt* mice. For C, E, H and J, student’s *t* test, * p < 0.05. **K** Representative sweeps with inter-stimulus interval of pair-pulse stimulations at 100 ms. Scale bars: 50 ms and 100 pA. **L** Paired-pulse ratios plotted against inter-stimulus intervals. n = 16 neurons from 4 mice for both genotypes. Two-way ANOVA, p = 0.074. **M** Depressed I/O curves in the hippocampus of *mt* mice. fEPSPs were recorded by stimulating the SC-CA1 pathway with gradual increasing intensities. *n* = 9 neurons from 4 *wt* mice, *n* = 10 neurons from 3 *mt* mice. Two-way ANOVA, p < 0.0001. **N** Impaired LTP at SC-CA1 pathway in the *mt* mice. Representative traces shown fEPSP indicated with 1 and 2. Scale bars represent 2 ms, 0.5 mV. **O** The maximum evoked fEPSP slope is measured during the first 10 min after induction. **P** The enhanced fEPSP slope is measured during the last 20 min of recording. For O and P, Student’s *t* test, * p < 0.05

To determine whether DGCR2 regulates synaptic transmission, we measured miniature excitatory postsynaptic currents (mEPSCs) in CA1 pyramidal neurons of the hippocampus (Fig. 3A). Recording was performed in the presence of TTX to block action potentials and bicuculine to block GABA transmission. As shown in Fig. 3D, E, no difference was observed in mEPSC amplitude between *wt* and *mt* slices. However, mEPSC frequency was decreased in *mt* hippocampus (Fig. 3B, C), suggesting that DGCR2 is required for synapse formation or plasticity. We also recorded miniature inhibitory postsynaptic currents (mIPSCs) in CA1 pyramidal neurons (Fig. 3F). Neither the frequency nor amplitude of mIPSC was changed in *mt* slices (Fig. 3G-J). These results suggest that glutamate release or functional synapse number may be impaired in *Dgcr2 mt* mice.

To determine whether the reduced mEPSC frequency in *Dgcr2 mt* mice is due to a change in glutamate release probability, we measured field excitatory postsynaptic potentials (fEPSPs) evoked by two presynaptic stimulations delivered at 25 ms intervals (i.e., paired pulses). No difference was observed in paired-pulse facilitation (PPF) of fEPSPs between *wt* and *mt* slices, suggesting glutamate release probability was not impaired in *Dgcr2 mt* mice (Fig. 3K, L).

As the changes in dendritic spine density are associated with synaptic plasticity [29], we further determined if DGCR2 regulates hippocampal long-term potentiation (LTP). fEPSPs were recorded by stimulating the Schaffer collateral (SC) - CA1 pathway with gradually increasing intensities. As shown in Fig. 3M, the I/O curves were depressed in *Dgcr2 mt* mice. We induced LTP in SC using high-frequency stimulation and found that the maximum fEPSP slope in the initial 10 min and the enhanced fEPSP slope in the last 20 min were reduced in *Dgcr2 mt* mice (Fig. 3N-P). These results suggest that LTP is impaired in *Dgcr2 mt* mice.

### Interaction of DGCR2 with NRXN1

**Fig. 4:**
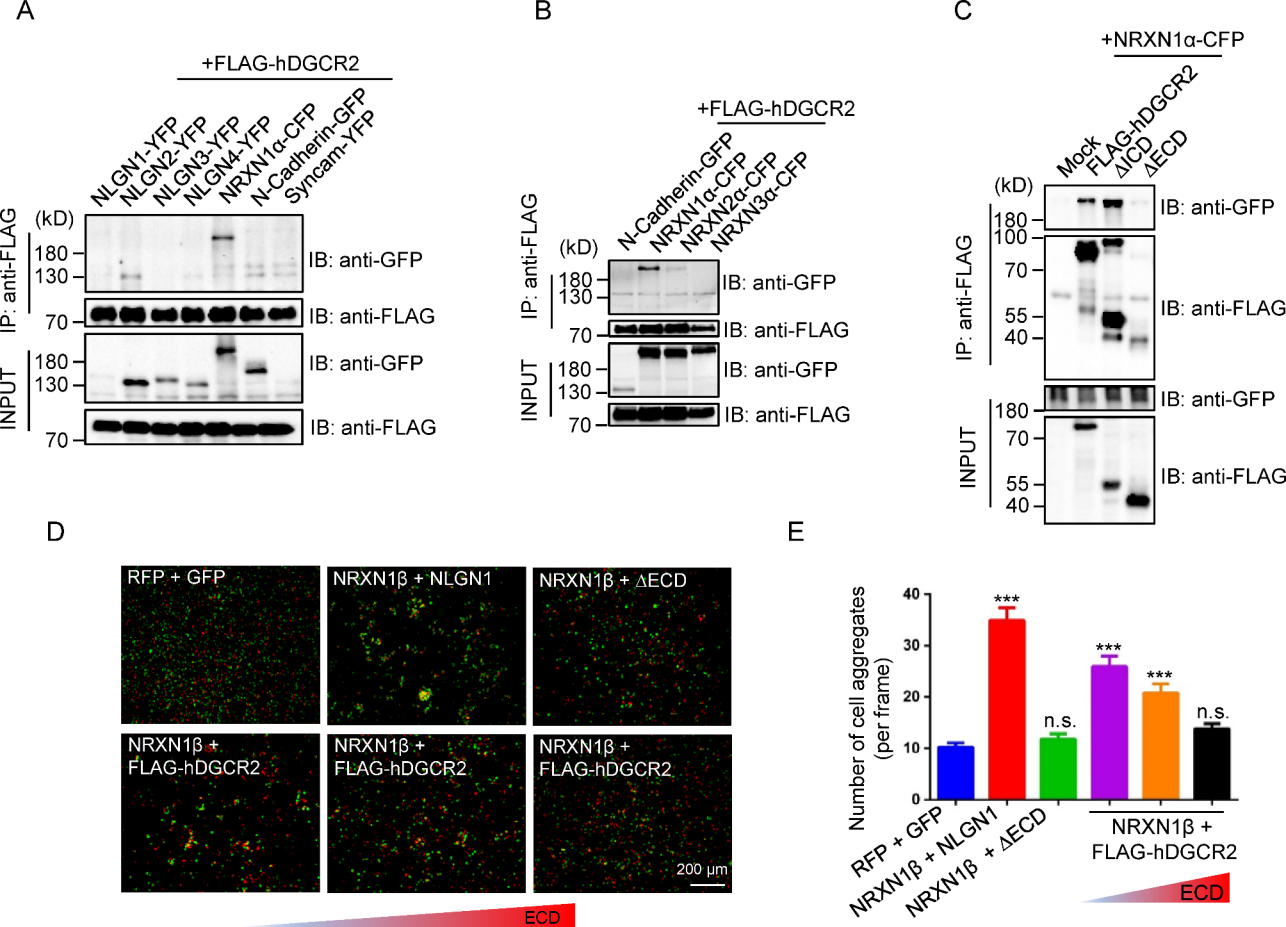
Interaction of DGCR2 with NRXN1. **A** DGCR2 interacts with NRXN1α in HEK 293T cells. Cells were co-transfected with FLAG-hDGCR2 and indicated constructs. Lysates were immunoprecipitated with anti-FLAG antibody and resulting complexes were blotted with anti-GFP and anti-FLAG antibodies. Inputs were blotted for perspective proteins as controls. **B** Surface DGCR2 interacts with NRXN1α in HEK 293T cells. Cells were co-transfected with FLAG-hDGCR2 and indicated constructs. Before lysis, the anti-FLAG antibody was added to the cells for intact cells IP. **C** ECD of DGCR2 interacts with NRXN1α in HEK 293T cells. Cells were co-transfected with NRXN1α-CFP and FLAG-hDGCR2, ΔICD, ΔECD or empty vector (Mock). Lysates were immunoprecipitated with anti-FLAG antibody and resulting complexes were blotted with anti-GFP and anti-FLAG antibodies. Inputs were blotted for perspective proteins as controls. **D-E** ECD of DGCR2 mediates its transcellular interaction with NRXN1 in HEK 293T cells. HEK 293T cells expressing NRXN1β or Mock along with RFP (red cells) were mixed with HEK 293T cells expressing NLGN1, Mock, FLAG-hDGCR2 or ΔECD along with GFP (green cells), respectively. An increasing amount of soluble ECD (0, 100 and 200 µL, respectively) was added to the cell mixtures of NRXN1β-expressing cells (red cells) and DGCR2-expressing cells (green cells). Cells were imaged, and aggregates were counted. Scale bar, 200 μm. From left to right in **E**, n = 12, 11, 10, 12, 9 or 10 images from three independent experiments. *** p < 0.001. n. s., p > 0.05, One-way ANOVA

It has been reported that DGCR2 was a putative adhesion receptor protein when its gene was discovered [23], and we found DGCR2 was localized to the PSDs. We hypothesized that DGCR2 may interact with a cell adhesion molecule to promote synapse formation. So, we tested if there are interactions between DGCR2 and other cell adhesion molecules in HEK 293T cells. As shown in Fig. 4A, Neurexin1α (NRXN1α) -CFP was co-immunoprecipitated with FLAG-hDGCR2, while Neurolign (NLGN) 1, 2, 3, 4-YFP and N-Cadherin-GFP weren’t. To further detect whether surface DGCR2 interacts with NRXNs, we confirmed the interaction between DGCR2 and other NRXNs through immunoprecipitation (IP) in intact cells. The anti-FLAG antibody was added directly to transfected cells before lysis to bind cell surface FLAG-hDGCR2. As shown in Fig. 4B, the interaction of FLAG-hDGCR2 with NRXN1α-CFP was much stronger than with NRXN2α-CFP. And FLAG-hDGCR2 didn’t interact with NRXN3α-CFP. Moreover, the ECD deletion mutant of FLAG-hDGCR2 (ΔECD) abolished its interaction with NRXN1α-CFP, while the ICD deletion mutant (ΔICD) didn’t (Fig. 4C). To confirm the DGCR2-NRXN1 interaction was transcellular, we did the cell aggregation assay in transfected HEK 293T cells. As a positive control, NRXN1β-expressing cells (red, co-expressing RFP) formed large aggregates with NLGN1-expressing cells (green, co-expressing GFP) (Fig. 4D-E). Similarly, FLAG-hDGCR2-expressing cells (green) also formed large aggregates with NRXN1β-expressing cells (red), while ΔECD-expressing cells (green) didn’t (Fig. 4D-E). Furthermore, we generated secretable ECD expression construct containing the entire FLAG-hDGCR2-ECD fused to an Fc fragment. ECD was purified from the conditional medium (CM) of transfected HEK 293T cells (Additional file 4: Figure S4) and added into the medium of the cell aggregation assay to neutralize the interaction between FLAG-hDGCR2 and NRXN1β. As shown in Fig. 4D-E, increasing amounts of ECD gradually disrupted transcellular interaction between FLAG-hDGCR2-expressing cells (green) and NRXN1β-expressing cells (red). These results suggest that the ECD of DGCR2 mediates its transcellular interaction with NRXN1.

### Regulation of dendritic spine formation by DGCR2-NRXN1 interaction

**Fig. 5:**
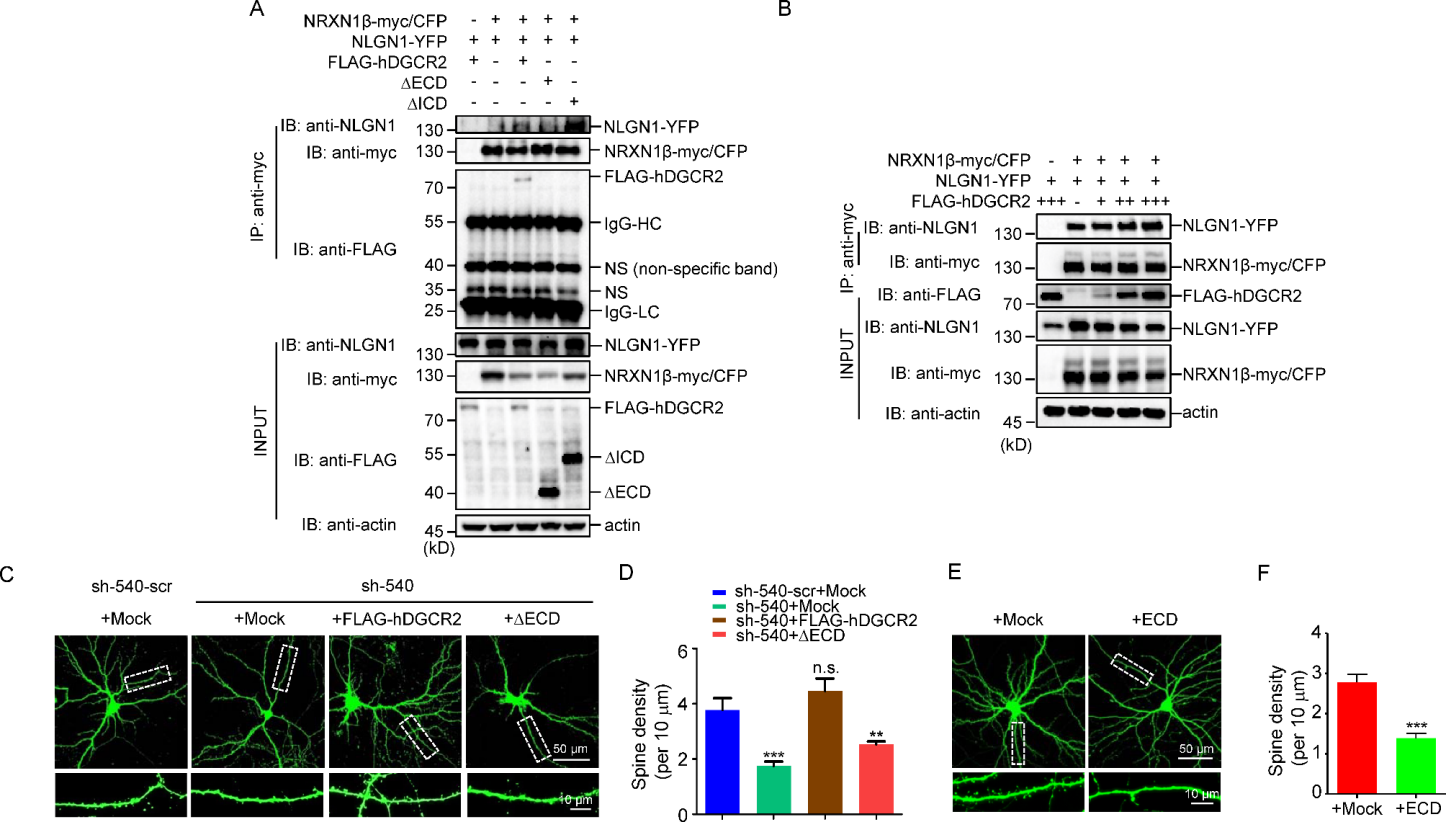
Regulation of dendritic spine formation by DGCR2-NRXN1 interaction. **A** DGCR2 promotes NRXN1β–NLGN1 interaction through its ECD. HEK 293T cells were co-transfected with NRXN1β-myc/CFP, NLGN1-YFP and FLAG-hDGCR2, ∆ECD or ∆ICD. Lysates were immunoprecipitated with anti-myc antibody and Protein A/G agarose, and resulting complexes were blotted with anti-NLGN1, anti-myc and anti-FLAG antibodies. Inputs were blotted for perspective proteins as control. Actin served as a loading control. IgG-HC, IgG heavy chain; IgG-LC, IgG light chain; NS, non-specific band. **B** HEK 293T cells were co-transfected with NRXN1β-myc/CFP, NLGN1-YFP, and an increasing amount of FLAG-hDGCR2. Lysates were immunoprecipitated with anti-myc antibody and Protein A/G agarose, and resulting complexes were blotted with anti-NLGN1, anti-myc and anti-FLAG antibodies. Inputs were blotted for perspective proteins as control. Actin served as a loading control. **C-D** ΔECD mutant of DGCR2 cannot rescue reduced spine density caused by DGCR2 knockdown. Primary hippocampal neurons were transfected at DIV 9 with indicated constructs and fixed for staining at DIV 16. Representative images of neuronal morphology (upper panel) and dendrite spines (lower panel) of cultured hippocampal neurons. Scale bars as indicated. Spine density (per 10 μm) quantitative analysis of data in C (**D**). n = 18 neurons for each condition. ** p < 0.005, *** p < 0.001. n. s., p > 0.05, One-way ANOVA. **E-F** DGCR2-ECD treatment impairs spine development. HEK 293T cells were transfected with secreted FLAG-hDGCR2-ECD or its mock construct. The conditional medium (CM) of transfected HEK 293T cells was collected and added to the primary hippocampal neurons at DIV 9 ~ 10 for 3 ~ 4 days. Representative images of neuronal morphology (upper panel) and dendrite spines (lower panel) of cultured hippocampal neurons (**E**). Scale bars as indicated. Spine density (per 10 μm) quantitative analysis of data in E (**F**). n = 10 neurons for each condition. *** p < 0.001

Postsynaptic NLGNs are classic binding partners for presynaptic NRXNs; this transsynaptic interaction is critical for synapse formation [30]. DGCR2 regulated dendritic spine formation and interacted with NRXN1transcellularly through its ECD, so we wonder if DGCR2 contributes to the NRXN1-NLGN1 interaction. We co-transfected DGCR2, ΔECD, ΔICD, or empty vector (Mock) with NRXN1β and NLGN1 in HEK 293T cells. By co-IP assay, we found that more NLGN1 was co-IPed by NRXN1β when DGCR2 or ΔICD, other than Mock or ΔECD, was introduced (Fig. 5A). These results suggest that DGCR2 facilitates NRXN1-NLGN1 interaction. And this facilitation was in a dose-dependent of DGCR2 (Fig. 5B).

As the ECD of DGCR2 is responsible for the DGCR2-NRXN1 interaction and its facilitation of NRXN1-NLGN1 interaction, we tested ECD’s role in spine formation. We overexpressed FLAG-hDGCR2 or ΔECD in DGCR2 knockdown neurons. As shown in Fig. 5C and D, overexpressing FLAG-hDGCR2 rescued the reduced spine density in sh-540 transfected neurons, while overexpressing ΔECD didn’t. Moreover, we examined spine development after neutralizing endogenous NRXN1-DGCR2 interaction. We collected the CM from HEK 293T cells transfected with ECD or control vector and added them to the hippocampal neurons transfected with GFP. Compared with the control, the spine density is reduced upon ECD treatment (Fig. 5E and F). These results suggest that inhibition of DGCR2-NRXN1α interaction disrupted spine development, indicating this interaction promotes dendritic spine formation.

### Anxiety-like behaviors in ***Dgcr2 mt*** mice

**Fig. 6:**
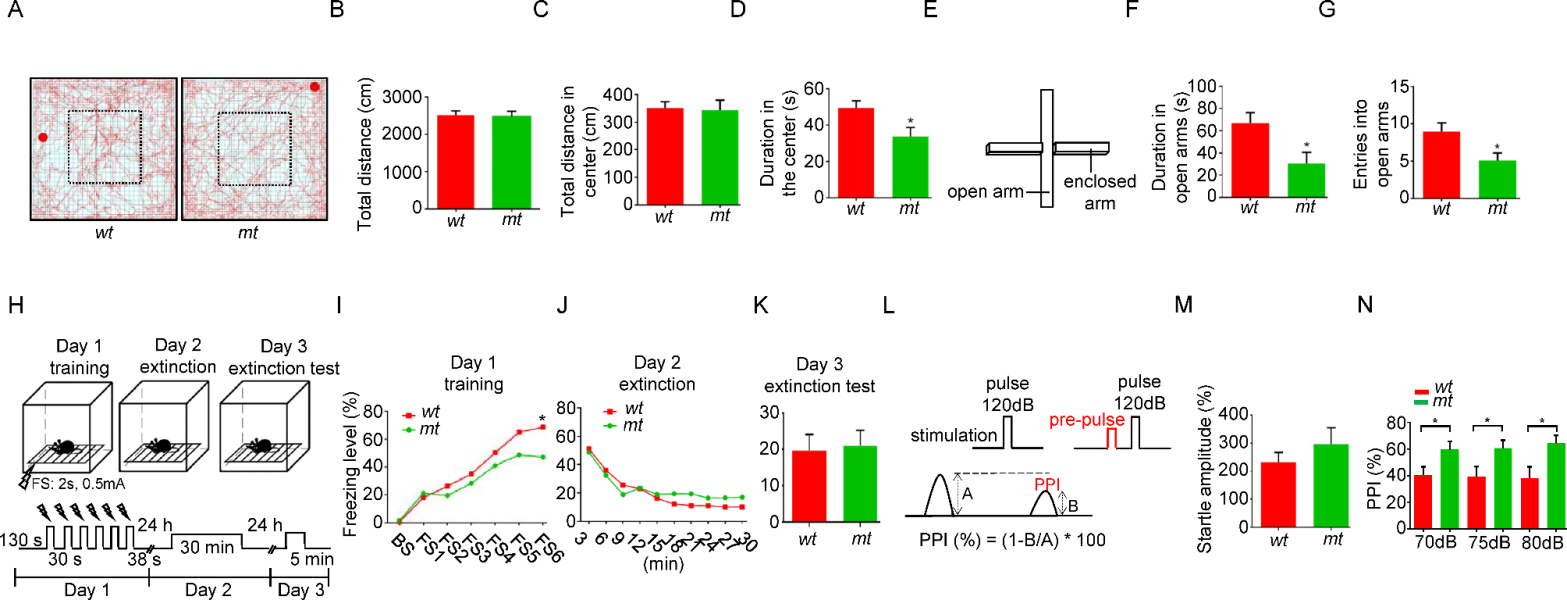
Abnormal behaviors in *Dgcr2 mt* mice. **A-D** Open field test (OFT). Representative traces of mice in the OFT (**A**). Mice were placed in the chambers and movement was monitored for 10 min. The square with dotted line indicates the center area. Distances traveled in total (**B**) or center area (**C**) during 10 min was similar between *wt* and *mt* mice. Decreased movement time in center area in *mt* mice (**D**). n = 21 *wt* mice and 14 *mt* mice. **E-G** Elevated plus maze test (EPM). Diagram of EPM (**E**). Mice were put in the center of EPM and they can freely explore for 10 min. The time stayed in the open arms (**F**) and entries into the open arms (**G**) were recorded. n = 21 *wt* mice and 14 *mt* mice. **H-K** Contextual fear conditioning test (contextual FC). Diagram of contextual FC (**H**). Day 1, six foot-shocks (FS) were delivered (0.5mA, 2s) during training. Day 2 and day 3, mice were placed into the same chambers for 30 min and 5 min, respectively, and the freezing time was recorded. BS, baseline. Impaired fear acquisition in *Dgcr2 mt* mice after five FS (**I**). Similar fear extinction between *mt* and *wt* (**J-K**). n = 13 *wt* mice and 12 *mt* mice. **L-N** Prepulse inhibition test (PPI). Diagram of PPI (**L**). Response to auditory-evoked startle stimulus (120 dB) was measured (**M**). The *mt* and *wt* exhibited similar baseline of startle responses. Increased PPI in *mt* mice (**N**). n = 12 *wt* mice and 11 *mt* mice. * p < 0.05, Student’s *t* test

The above results demonstrate that DGCR2 is a cell adhesion molecule regulating dendritic spine formation. We wonder if the *Dgcr2 mt* mice exhibit any abnormal behaviors. Compared to *wt*, *mt* mice spent less time in the central area of the open field test (OFT) (Fig. 6A-D). And in the elevated plus maze (EPM), *mt* mice entered less and spent less time in the open arms (Fig. 6E-G). These results suggest that *Dgcr2 mt* mice exhibit anxiety-like behaviors.

As DGCR2 regulated spine formation and glutamatergic transmission in the hippocampus, we characterized the effects of *Dgcr2* mutation on hippocampus-related behaviors. *Mt* mice were subjected to contextual fear conditioning (FC) (Fig. 6H), a hippocampus-dependent behavioral paradigm, to test associative memory formation and consolidation. During training, *mt* mice showed worse context freezing acquisition than *wt* mice (Fig. 6I). However, the freezing time of *mt* was similar to *wt* when reintroduced into the same cage during the extinction and extinction test (Fig. 6J-K). These results indicate that *Dgcr2* deficiency inhibits fear acquisition.

Prepulse inhibition (PPI) of the startle reflex is a behavioral paradigm to test the sensory-motor gating which is often reduced in schizophrenics [31, 32]. A combination of an auditory-evoked startle stimulus (120 dB) and three different levels of prepulse stimuli (70, 75 and 80 dB) was applied to measure PPI (Fig. 6L). The *mt* mice showed an increase, but not significant, in startle responses (Fig. 6M), and interestingly, PPI was substantially increased in *mt* mice (Fig. 6N). Taken together, these results suggest that *Dgcr2 mt* mice exhibit abnormal behaviors.

## Discussion

Here we provide evidence that the expression of *DGCR2*, a schizophrenia risk gene, increased during neurodevelopmental stages and was enriched in the PSDs. Knockdown of DGCR2 in cultured neurons and mutation in mice reduced the dendritic spine density of hippocampal neurons. mEPSC frequency was decreased in the hippocampal neurons of *Dgcr2 mt* mice, and hippocampal LTP was also impaired. In vitro data indicate that the ECD of DGCR2 is responsible for its transcellular interaction with the cell adhesion molecule NRXN1. DGCR2-NRXN1 interaction promotes NRXN1-NLGN1 binding and dendritic spine development. Behaviorally, *mt* mice exhibited anxiety-like behaviors, impaired fear acquisition, and increased PPI. Together, these results demonstrate that DGCR2 plays a critical role in regulating dendritic spine development, thus revealing potential pathophysiological mechanisms of 22q11DS and related mental disorders.

*DGCR2* is one of the deleted genes within 22q11.2 deletion, which is a strongest genetic risk factor for schizophrenia. In mice models for 22q11DS, *Df(16)A* and *LgDel* mice are with the largest deletion including *Dgcr2* [19, 33]. In these two mice, spine density is decreased in the hippocampal CA1 neurons [34, 35]. The frequency of mEPSCs of hippocampal neurons in *Df(16)A* mice is reduced, but the amplitude is not altered [35]. These deficits were also observed in *Dgcr2 mt* mice.

Surprisingly, the PPI of the startle response was increased in *Dgcr2 mt* mice, which phenotype is in line with the *DelAwb* mice with minimal 22q11.2 deletion including *Dgcr2* [36]. Usually, PPI is reduced in schizophrenia patients and mice models [31]. However, there are also some mice models of neuropsychiatric disorders that exhibit increased PPI, like schizophrenia-related Neurolign2 R215H knockin mice [37] and autism spectrum disorder (ASD)-related *Shank3* knockout mice (*Shank3*^*tm2Gfng*^) [38]. PPI reflects the sensory-motor gating, and the underlying neural circuit includes the prefrontal cortex, nucleus accumbens, ventral pallidum, and pontine tegmentum [39, 40]. DGCR2 may have potential roles in these brain regions. Moreover, in the PPI test, the baseline startle response of *Dgcr2 mt* mice was increased, although the increase wasn’t significant in statistics. This increase implies that *Dgcr2 mt* mice may have abnormal hearing and acoustic startle reflex, thus contributing to the increased PPI.

Cell adhesion molecules in synapses regulate synapse formation and plasticity [41]. Especially in synapse formation, trans-synaptic adhesion proteins usually recruit pre- and postsynaptic membrane or cytoplasmic proteins to promote synapse formation. NRXNs are presynaptic transmembrane adhesion molecules interacting with postsynaptic NLGNs to regulate synapse specification, establishment, maturation and transmission [42–44]. Not only to NLGNs, NRXNs also bind to other cell adhesion molecules, like LRRTM [45], neurexophilin [46], dystroglycan [47] and so on. Here we found DGCR2 is a novel binding partner of NRXN11. This interaction is mediated by the ECD of DGCR2. Considering DGCR2 is localized to the PSDs, DGCR2 may interact with NRXN1 to form a trans-synaptic complex to regulate spine formation. And rescue experiments with ΔECD, as well as ECD treatments on spine density indicated the DGCR2-NRXN1 interaction is critical for spine development. Our findings provide mechanistic insight into the pathophysiological roles of *DGCR2* in 22q11DS and related mental disorders.

## Materials and methods

### Reagents, antibodies, and plasmids

Chemicals were purchased from Sigma-Aldrich unless otherwise indicated. Information of primary antibodies is as follows: mouse anti-FLAG (Sigma, F1804, 1:5000 for WB), mouse anti-GFP (Santa Cruz, sc-9996, 1:1000 for WB), mouse anti-GFP (Invitrogen, A-11,120, 1:1000 for staining), mouse anti-PSD95 (Millipore, MAB1598, 1:1000 for WB and 1:500 for staining), mouse anti-synaptophysin (Dako, M7315, 1:5000 for WB), rabbit anti-β-actin (Santa Cruz, sc-1616-R, 1:1000 for WB), mouse anti-Tau-1 antibody (Millipore, MAB3420, 1:500 for staining), mouse anti-MAP2 antibody (Millipore, MAB3418, 1:500 for staining), DGCR2 antibody was generated against hDGCR2-ICD in rabbit (1:1000 for WB and 1:200 for staining).

To generate FLAG-hDGCR2, the human *DGCR2* cDNA encoding 22–550 amino acids of DGCR2 without signal peptide was amplified by PCR and subcloned into pFLAG-CMV1 (Sigma, E7273) downstream of an artificial signal peptide sequence and a FLAG epitope. Different cDNAs encoding ΔECD and ΔICD of DGCR2 were amplified with primers 5’- GAAGATCTGATGCGCCTGGTCGTC-3’ and 5’- ACGCGTCGACCTACACCACAGTATTG-3’, 5’-GAAGATCTGCGGCCAGAGCTG-3’ and 5’- ACGCGTCGACCTACCGGTGGACCATGAAG-3’, and subcloned into pFLAG-CMV1 separately. NRXNs and NLGNs constructs were obtained as described previously [48]. The authenticity of all constructs was verified by DNA sequencing and western blotting analysis.

### Animals

All mice were housed in temperature-fixed (22 ± 2°C), humidity-controlled chambers, and sufficient food and water were administered daily. No more than 5 adult mice per cage were subjected to a 12-h light/dark cycle under standard conditions. All the mice were guaranteed to be hygienic. The animal experiments followed the “Guidelines for the Care and Use of Laboratory Animals” promulgated by Nanchang University. *Dgcr2*-LacZ mice were derived from mutant embryonic stem (ES) cells obtained from EUCOMM (stock#: 23939). In *Dgcr2*-LacZ mice, a cassette containing LacZ was inserted between exons 1 and 2 of the *Dgcr2* gene (Additional file 2: Figure S2A) (Skarnes et al., 2011). In addition, the polyadenylation termination signal contained in the cassette severely reduces the transcription of downstream DNA. Genotyping primers for the wild-type allele (*wt*) were: 5’-TGACTCTGGTGTCACCTCACTTCG-3’ and 5’-CCTGAGTCAGCCATTCCTGCTTCC-3’ (407 bp), and for the mutant allele (*mt*) were: 5’-TGACTCTGGTGTCACCTCACTTCG-3’ and 5’-CAACGGGTTCTTCTGTTAGTCC-3’ (340 bp).

### shRNA interference

For gene knockdown by RNA interference (RNAi), pSUPER vector (OligoEngine) – based small hairpin RNAs (shRNAs) of rat or mouse *Dgcr2*, *Dgcr2*-scramble were constructed. The shRNA target sense sequences for *Dgcr2* (sh-540 and sh-768) and *Dgcr2*-scramble (sh-540-scr) were 5’-ctgggttggttatcagtat-3’, 5’-gtcgtcatttctgtgtaaa-3’ and 5’-GGTTCTGACGTTGTAAGTT-3’.

### X-Gal assay

As described previously [49], mice were anesthetized and decapitated. Brain samples were isolated and rapidly frozen in OCT and cut into 40-µmsections and mounted on Super Frost Plus slides (Fisher). Sections were fixed for 2 min in a buffer containing (in millimoles): 2 MgCl_2_, 5 EGTA with 0.2% glutaraldehyde, and 2% (wt/vol) paraformaldehyde. Sections were washed in ice-cold phosphate-buffered saline (PBS) and stained in X-gal solution [1 mg/mL X-gal, 5 mM K_3_Fe(CN)_6_, 5 mM K_4_Fe(CN)_6_, 0.02% Nonidet P-40, 0.01% deoxycholate, and 2 mM MgCl_2_ in PBS] at 37 °C overnight. After washing with PBS, slices were counterstained with nuclear Fast Red (Vector Laboratories), mounted in Hydromount (National Diagnostics), and sealed with coverslips.

### PSD fractionation

The PSDs fraction was prepared as described previously [48, 50, 51]. Briefly, mouse brains were homogenized in HEPES buffer (0.32 M sucrose, 4 mM HEPES [pH 7.4]). The homogenate (Hom.) was centrifuged to remove the pelleted nuclear fraction (P1), and the supernatant (S1) was centrifuged again to yield the crude synaptosomal fraction (P2). The washed P2 fraction (P2’) was subjected to hypoosmotic shock and lysis before centrifugation again. After centrifugation, the supernatant (S3) was centrifuged to yield the pellet enriched with synaptic vesicle protein (SV fraction); and the resultant pellet (P3) was resuspended and centrifuged in a sucrose gradient to yield the synaptic plasma member (SPM) fraction. The SPM fraction was incubated with 1% Triton X-100 in 50 mM HEPES (pH 8.0) at 4 °C for 30 min and subjected to centrifugation to yield the supernatant (presynaptic membrane fraction, Pre) and the pellet (postsynaptic density, PSD).

### Cell culture and transfection

Human embryonic kidney (HEK) 293T cells were cultured in Dulbecco’s modified Eagle’s medium (DMEM) (Gibco) supplemented with 10% fetal bovine serum (FBS) (Gibco). Transient transfection was performed using polyethylenimine (Sigma, 408,727), as described before (Tao Yanmei, 2013 Nat Neurosci). Briefly, cells were cultured in 100 mm dishes and at ∼70% confluence were incubated with precipitates formed by 5 µg of plasmid DNA and 280 µL of polyethylenimine 0.05% (wt/vol). Cells were harvested 24 ~ 48 h posttransfection.

Cultures of primary hippocampal neurons were prepared from embryonic day (E) 18 Sprague–Dawley rats as described previously [48, 52]. Briefly, hippocampi were isolated and kept separate from one another in HBSS on ice. Following digestion in 0.25% trypsin plus 0.1 mg/mL DNase I (one hippocampus in 1 mL) at 37 °C for 20 min. Dissociated cells were resuspended in plating media (DMEM supplemented with 10% FBS) and plated at a density of 1 × 10^5^ or 2 × 10^5^ per well onto poly-D-lysine–coated 20-mm coverslips (WHB) in 12-well plates (Corning). Cells were incubated for 4 h before replacing with maintenance medium [neurobasal medium (Gibco) supplemented with 2% B-27 supplement (Gibco), 1% GlutaMax (Gibco), and 1% penicillin/streptomycin (Gibco)]. Neurons were maintained at 37 °C in 5% CO2, with half of the medium changed every 2–3 d.

To detect shRNA knockdown efficiencies in neurons, cortical neurons at 0 day in vitro (DIV) were transfected with indicated shRNA plasmids using 4D-Nucleofector (Lonza AG) according to the manufacturer’s protocol. To observe neuronal morphology, hippocampal neurons at DIV 7 ~ 9 were transfected with different plasmids plus enhanced GFP using calcium phosphate precipitation as described previously [48, 52]. Briefly, the neurons were serum-starved with pre-heated DMEM for 2 h at 37 °C in 10% CO_2_. For each well of 12-well plate, 1–6 µg DNA in 1–6 µL was mixed with 5 µL 2.5 M CaCl_2_ in ddH2O (total volume 50 µL), and further mixed with 50 µL of Hepes-buffered saline containing (in millimoles): 274 NaCl, 10 KCl, 1.4 Na_2_HPO_4_, 15 glucose, and 42 Hepes, pH 7.05. Resulting DNA-calcium phosphate precipitates were added into neurons. Morphology was studied 3–7 d later.

### ***In-utero*** electroporation

Pregnant mice at E14.5 or E15.5 were anesthetized, and subjected to an abdominal incision to expose the uterus. Adjust the embryo to a suitable position and using a beveled glass capillary injected manually 1 to 2 ml of DNA solution (final concentration 1 mg/ml) into the lateral ventricle of the embryos. The embryo’s brain was then exposed to electric pulses (five 50 ms, 36 v pulses at an interval of 1 s) using an electroporator (BTX, ECM830). Then, the uterus was placed back into the abdominal cavity before the wound surgically sutured. Embryos were allowed to develop normally, and positive pups were sacrificed at P30. The brain was fixed overnight in 4% paraformaldehyde in PBS, pH7.4 and cut into slices at 100 μm using Leica vibratome cutting system. The slices were subjected to the Olmpus FSX100 with Z stack imaging analyses.

### IP and WB

For co-immunoprecipitation (co-IP), transfected HEK 293T cells were lysed in IP buffer containing (in millimoles): 20 Tris, pH7.6, 50 NaCl, 1 EDTA, 1 NaF, 0.5% Nonidet P-40 (vol/vol), with protease and phosphatase inhibitors. Samples were centrifuged at 12,000 × g for 20 min at 4 °C to remove debris. Lysates (1–2 mg) were incubated with corresponding antibody (1–2 µg) at 4 °C for either 3–4 h or overnight and then incubated with 10–15 µL Protein A/G magnetic agarose beads (Pierce) at 4 °C for 1 h. Samples were washed with IP buffer and resuspended in SDS sample buffer. Then the samples were subjected to WB.

For intact cells IP, cells were washed with PBS, and the anti-FLAG antibody was directly added to the dish for 2–4 h incubation at 4 °C. Then the unbound antibody was washed with PBS, and cells were subjected to lysis in the IP buffer. After centrifuge at 12,000 × g for 20 min at 4 °C, the supernatants were added with 10–15 µL Protein A/G magnetic agarose beads for incubation at 4 °C for 1 h. Samples were washed with IP buffer and resuspended in SDS sample buffer. Then the samples were subjected to WB.

For protein expression detection, tissues were homogenized in PBS plus protease and phosphatase inhibitors. Then the homogenates were lysed in equal volumes of 2 × RIPA buffer [0.2% SDS (wt/vol), 1% sodium deoxycholate (wt/vol) and 2% Nonidet P-40 (vol/vol) in PBS] plus protease and phosphatase inhibitors. Lysates were centrifuged at 12,000 × g for 20 min at 4 °C to remove debris. The supernatants were subjected to Bradford assay (Pierce) to measure protein concentration and diluted in SDS sample buffer.

Protein samples (10–20 µg) were resolved by SDS-PAGE and transferred to PVDF membrane (Millipore). The membrane was immunoblotted with primary and secondary antibodies, and immunoreactive bands were visualized by enhanced chemiluminescence under the gel documentation system (Bio-Rad). Densitometric quantification of protein band intensity was performed by using ImageJ.

### Secretable DGCR2-ECD preparation

FLAG-tagged ECD of DGCR2 (aa 22–346, ECD of DGCR2 without signal peptide) was amplified from pFLAG-CMV1-hDGCR2 and subcloned into pcDNA/Fc [53] to generate FLAG-ECD-Fc (ECD) expression construct. As described previously [54], HEK 293T cells were transfected with ECD or its mock vector and 24 h later, cells were switched to the neurobasal medium (Gibco). Conditional medium (CM) containing secreted ECD or mock Fc were harvested 24 h later and added to the medium of primary hippocampal neurons transfected with GFP.

### Immunostaining

As described previously [55], primary cultured neurons were fixed with 4% paraformaldehyde/4% sucrose (wt/vol) for 15 min. After washing three times with PBS, neurons were incubated with primary antibody diluted in GDB buffer (30 mM phosphate buffer, pH 7.4, containing 0.2% gelatin, 0.6% Triton X-100, and 0.9 M NaCl) at 4 °C overnight. After washing three times with washing buffer (20 mM phosphate buffer and 0.5 M NaCl), neurons were incubated with the corresponding Alexa Fluor-conjugated secondary antibodies (diluted in GDB buffer) at room temperature for 1 h. Images were taken under a Olympus FV1000 scanning confocal microscope with a 60× oil immersion objective. Ten to 15 serial individual optical sections were collected (z interval of 0.5 μm). Dendritic spines were quantified by ImageJ: three isolated dendritic segments (50–60 μm long) that were about 20 μm away from the cell body were analyzed for each neuron. The quantification of spine density was performed in blinded fashion.

### Cell aggregation assay

HEK 293T cells were transfected with indicated expression constructs, respectively. As described previously [48], 48 h later, the cells were detached from the culture plates with 1 mM EDTA in PBS. Then the green cells were mixed with red cells in each individual condition as indicated in the figures and incubated with gentle agitation at room temperature in DMEM supplemented with 10% FBS, 50 mM Hepes-NaOH, pH 7.4, 10 mM CaCl_2_, and 1o mM MgCl_2_. After 1 h, the cell mixtures were gently transferred into a 12-well plate and imaged by fluorescence microscopy to assess the extent of cell aggregation. The resulting images were analyzed by counting the number of aggregation particles in the field using ImageJ. Cell aggregation particles were defined as a group of four or more clustered cells with at least one red and green cell.

### Golgi staining

Golgi staining was performed by using the FD Rapid GolgiStain Kit following the manufacturer’s protocol (FD NeuroTechnologies) as previously [48]. Brain tissues were incubated in mixed solutions A and B for 2 weeks in the dark at room temperature and put into solution C for 3 d. Tissues were cut into slices with 100-µm thickness, stained with solutions D and E, dehydrated in gradient ethanol, cleared with xylene, and mounted on slides for imaging. Images of pyramidal neurons in the hippocampal CA1 region were taken and imported into ImageJ for analysis. Spines of secondary and tertiary dendritic branches of randomly selected segments (20 μm each) of CA1 neurons were quantified.

### Electrophysiological analysis

Mice (5-7-week-old) were anesthetized with isoflurane and killed by decapitation. Brains were quickly removed to ice-cold oxygenated (95% O_2_/5% CO_2_) cutting solution containing (in millimoles): 120 Choline Chloride, 2.5 KCl, 7 MgCl_2_, 0.5 CaCl_2_, 1.25 NaH_2_PO_4_, 25 NaHCO_3_, and 10 glucose. Lamellar 300 μm slices of the hippocampus using VT1000S Vibratome (Leica Microsystems) as described elsewhere (Bischofberger et al., 2006). The slices were recovered in oxygenated artificial cerebrospinal fluid (ACSF) for 30 min at 32 °C and maintained at room temperature (25 ± 1 °C) for an additional 1 h before recording. The ACSF containing (in millimoles): 124 NaCl, 2.5 KCl, 2 MgSO_4_, 2.5 CaCl_2_, 1.25 NaH_2_PO_4_, 26 NaHCO_3_, and 10 glucose.

Slices were transferred to a recording chamber superfused (2 mL/min) with ACSF at 32–34 °C. Slices were visualized with infrared optics using an upright fixed microscope equipped with a 40 × water-immersion lens (FN-S2N, Nikon) and infrared CCD monochrome video camera (IR-1000, DAGE-MTI). The patch pipettes were pulled by a horizontal pipette puller (P-1000; Sutter Instrument) with a resistance of 3–5 MΩ. Recording were preformed with MultiClamp 700B amplifier and 1550 A digitizer (Molecular Device) at 32–34 °C. Series resistance was below 20 MΩ and monitored throughout the experiments.

For mEPSCs recording, pyramidal neurons were held at -70 mV in the presence of bicuculline (20 µM) and tetrodotoxin (TTX, 1 µM), with the pipette solution containing (in millimoles): 125 K-gluconate, 5 KCl, 10 HEPES, 0.2 EGTA, 1 MgCl_2_, 4 Mg-ATP, 0.3 Na-GTP and 10 phosphocreatine (pH 7.35, 285 mOsm).

For mIPSCs recording, pyramidal neurons were held at -70 mV in the presence of CNQX (20 µM), DL-2-amino-5-phosphonopentanoic acid (DL-AP5, 100 µM) and TTX (1 µM), with the pipette solution containing (in millimoles): 130 KCl, 10 HEPES, 0.2 EGTA, 1 MgCl_2_, 4 Mg-ATP, 0.3 Na-GTP and 10 phosphocreatine (pH 7.35, 285 mOsm).

For paired-pulse ratio recording, EPSCs were evoked by stimulating SC-CA1 pathway at holding potential of -70 mV in the presence of BMI (20 µM), with the pipette solution containing (in millimoles): 125 Cs-methanesulfonate, 5 CsCl, 10 HEPES, 0.2 EGTA, 1 MgCl_2_, 4 Mg-ATP, 0.3 Na-GTP, 10 phosphocreatine and 5 QX314 (pH 7.35, 285 mOsm). Interval of paired stimulations was set at 25 ms. Ratio was defined as the fraction of EPSC2/EPSC1 amplitudes. Data were filtered at 1 kHz and sampled at 10 kHz.

### Behavior analysis

Behavior analysis was carried out using 8–12 weeks-old male mice by investigators unaware of genotypes. Mice were handled by investigators for three to five days before each behavior test. And before the behavior tests, the mice should adapt to the experiment environment for 1 h.

OFT was measured as the previous description [50]. Mice were placed in a chamber (50 × 50 cm), and an overhead camera and tracking software (Med Associates) were used to monitor the mouse movement for 10 min.

EPM (Med Associates) was an anxiety-like behavior test. The platform was elevated 74 cm above the floor. It consists of two closed (35 × 6 × 22 cm), two open (35 × 6 cm) arms and a central zone (6 × 6 cm). Mice were placed on the central zone and faced an open arm. Mice can freely explore the platform for 10 min. The total time spent in the open arms and the entries to open arms were recorded by the monitoring software.

For the contextual FC test, fear training, fear extinction and extinction test were finished in three days. On the first day, mice were placed in the conditioning chambers for 330 s and exposed to foot shocks at 130 s, 162 s, 194 s, 226 s, 258 s, 290 s for 2 s, 0.5 mA. Each foot shock was 30 s interval. 24 h. later, mice were placed in the same chambers for 30 min without foot shocks for fear extinction. After 24 h. mice were tested in the same chamber for 5 min. All the freezing time were recorded by the monitor software.

Prepulse inhibition (PPI) was finished in sound-attenuated chambers (Med Associateds) as described previously [56]. Briefly, mice were allowed to habituate to the chamber for 5 min with 65 dB background white noise. During the test, mice were placed in a Plexiglass tube, then faced to a pseudorandomly mixed 12 startle trials (120 dB, 20 ms) and 12 prepulse/startle trials (70, 75, or 80 dB white noise for 20 ms,100 ms intervals and 120 ms startle stimulus for 20 ms). Two consecutive trials were not the same. Mouse movement was measured during 100 ms after startle stimulus onset (sampling frequency 1 kHz). PPI (%) was calculated according to the formula: 100 × (1 - startle amplitude on prepulse-pulse trials / startle amplitude on pulse alone trials).

## Electronic supplementary material

Below is the link to the electronic supplementary material.

Additional file 1: Figure S1. Expression of DGCR2 in mice. **A** DGCR2 was expressed in the brain and several peripheral tissues. Indicated tissues were collected from adult *wt* male mice and homogenized for WB. Actin served as a loading control. **B** DGCR2 was abundant in different brain regions. Tissues of indicated brain regions from adult *wt* male mice and homogenized for WB. Actin served as a loading control. **C** DGCR2 expression in the brain was regulated developmentally. The whole brain at indicated different stages were collected from *wt* mice and homogenized for WB. Actin served as a loading control.

Additional file 2: Figure S2. Characterization of *Dgcr2*-LacZ mice. **A** Schematic diagram of *Dgcr2*-LacZ mice genomic structure. **B** X-Gal staining of brain slices from adult male *Dgcr2*-LacZ homozygous mouse. Scale bar as indicated. **C-D** DGCR2 expression in the brain of *Dgcr2*-LacZ mice. Whole brains from adult male *Dgcr2*-LacZ homozygous mice (*mt*) or control *wt* mice were isolated and homogenized for WB. Actin served as a loading control. Representative blots (**A**) and quantification data (**B**). n = 3 mice for each genotype. * p < 0.05, Student’s *t* test.

Additional file 3: Figure S3. Knockdown efficiency of DGCR2 shRNAs in primary neurons. Rat primary cortical neurons on DIV0 were nucleofected with shRNAs of rat DGCR2 or control (empty vector) and harvested on DIV5 for WB. Actin served as a loading control. Representative blots (**A**) and quantification data (**B**). Data were from three independent experiments. * p < 0.05, One-way ANOVA.

Additional file 4: Figure S4. Preparation of secretable DGCR2-ECD. FLAG-hDGCR2-ECD-Fc (ECD) expression construct or empty FLAG-Fc construct (Mock) were transfected into HEK 293T cells. Cell lysates and conditional media (CM) were collected. To concentrate secreted ECD, the CM were subjected into IP with anti-FLAG antibody. IgG-HC indicates IgG heavy chain, and IgG-LC indicates IgG light chain.

## Data Availability

The datasets supporting the conclusions of this article are included within the article and its additional files. All data generated and analyzed in this study are available from the corresponding author on reasonable request.
