# Supplementary figures and images for: DiGeorge syndrome critical region gene 2 (DGCR2), a schizophrenia risk gene, regulates dendritic spine development through cell adhesion
Source: Cell Biosci. 2023 Jul 21;13:134. doi: 10.1186/s13578-023-01081-9 (PMC10362570; doi:10.1186/s13578-023-01081-9)

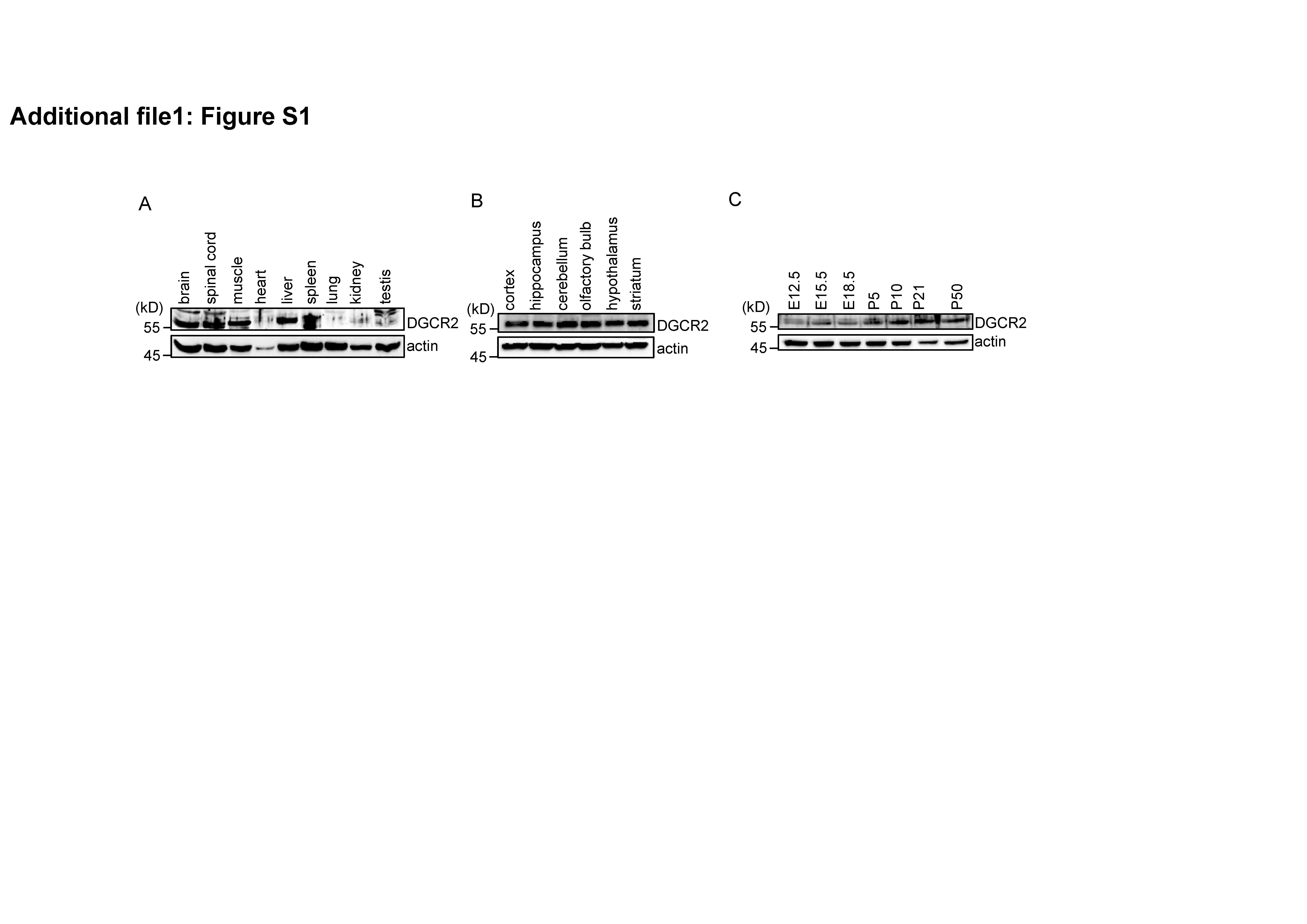

Supplement: Supplementary file 1 — Additional file 1: Figure S1. Expression of DGCR2 in mice. A DGCR2 was expressed in the brain and several peripheral tissues. Indicated tissues were collected from adult wt male mice and homogenized for WB. Actin served as a loading control. B DGCR2 was abundant in different brain regions. Tissues of indicated brain regions from adult wt male mice and homogenized for WB. Actin served as a loading control. C DGCR2 expression in the brain was regulated developmentally. The whole brain at indicated different stages were collected from wt mice and homogenized for WB. Actin served as a loading control. [file 13578_2023_1081_MOESM1_ESM.tiff]

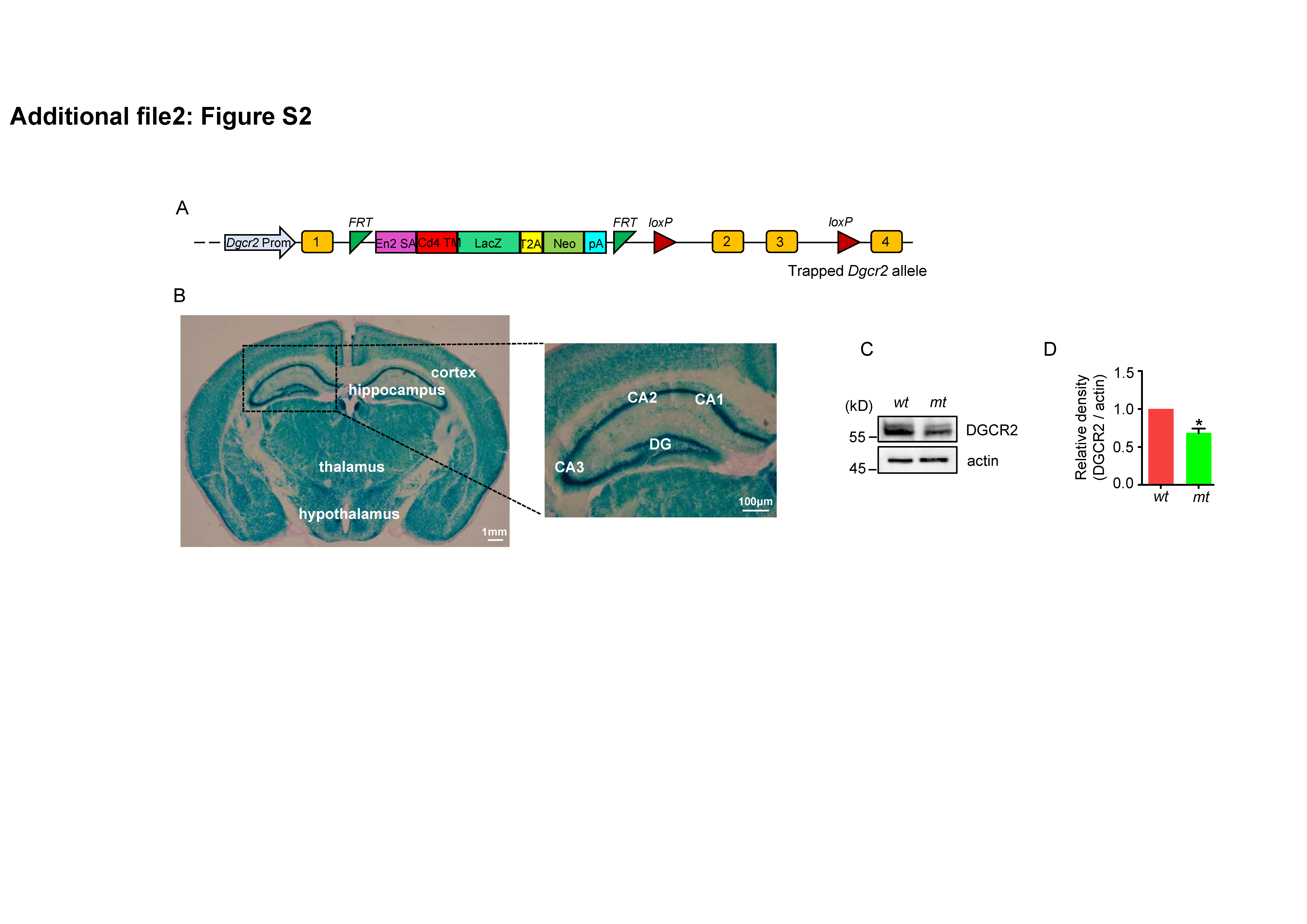

Supplement: Supplementary file 2 — Additional file 2: Figure S2. Characterization of Dgcr2-LacZ mice. A Schematic diagram of Dgcr2-LacZ mice genomic structure. B X-Gal staining of brain slices from adult male Dgcr2-LacZ homozygous mouse. Scale bar as indicated. C-D DGCR2 expression in the brain of Dgcr2-LacZ mice. Whole brains from adult male Dgcr2-LacZ homozygous mice (mt) or control wt mice were isolated and homogenized for WB. Actin served as a loading control. Representative blots (A) and quantification data (B). n = 3 mice for each genotype. * p < 0.05, Student’s t test. [file 13578_2023_1081_MOESM2_ESM.tiff]

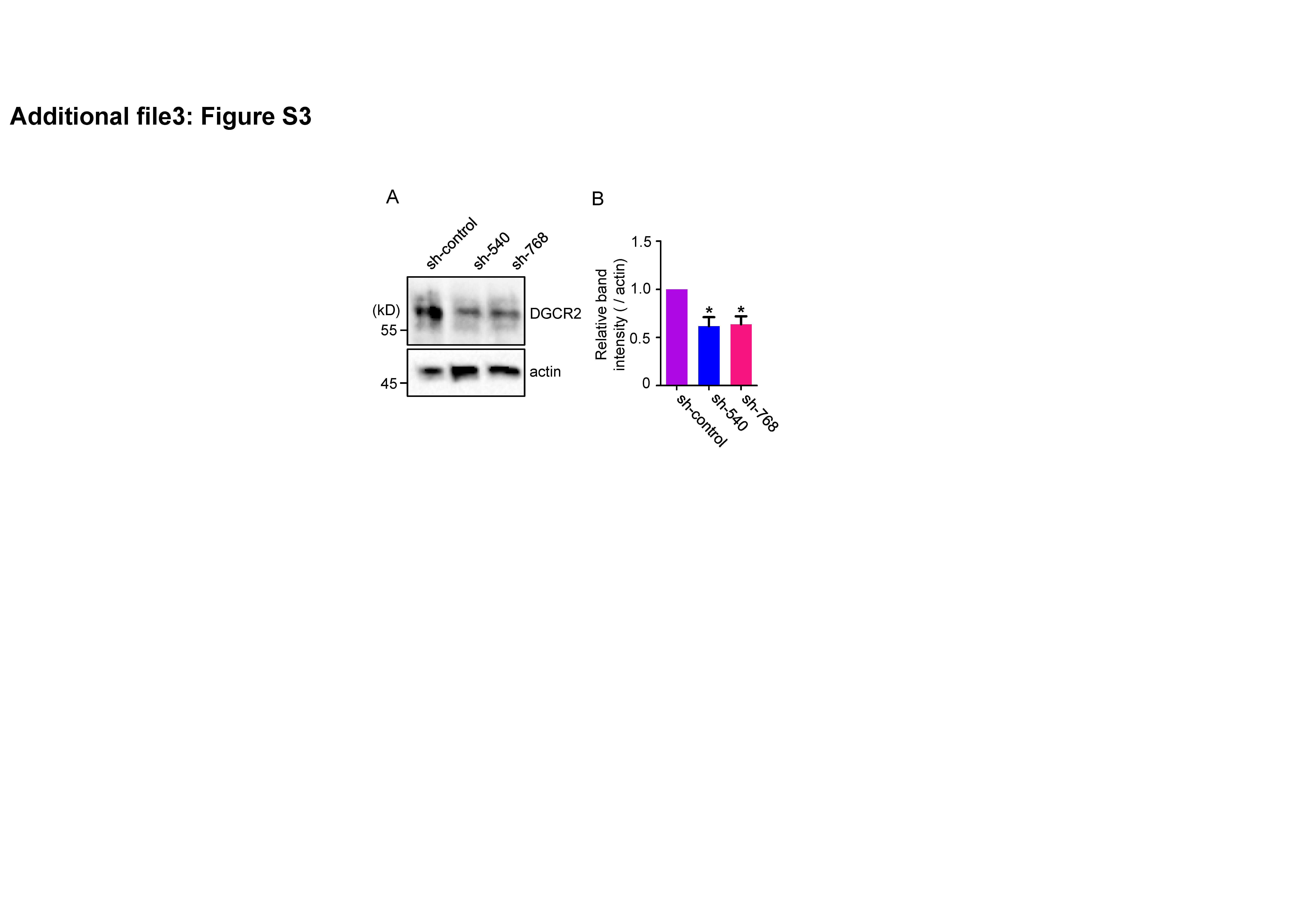

Supplement: Supplementary file 3 — Additional file 3: Figure S3. Knockdown efficiency of DGCR2 shRNAs in primary neurons. Rat primary cortical neurons on DIV0 were nucleofected with shRNAs of rat DGCR2 or control (empty vector) and harvested on DIV5 for WB. Actin served as a loading control. Representative blots (A) and quantification data (B). Data were from three independent experiments. * p < 0.05, One-way ANOVA. [file 13578_2023_1081_MOESM3_ESM.tiff]

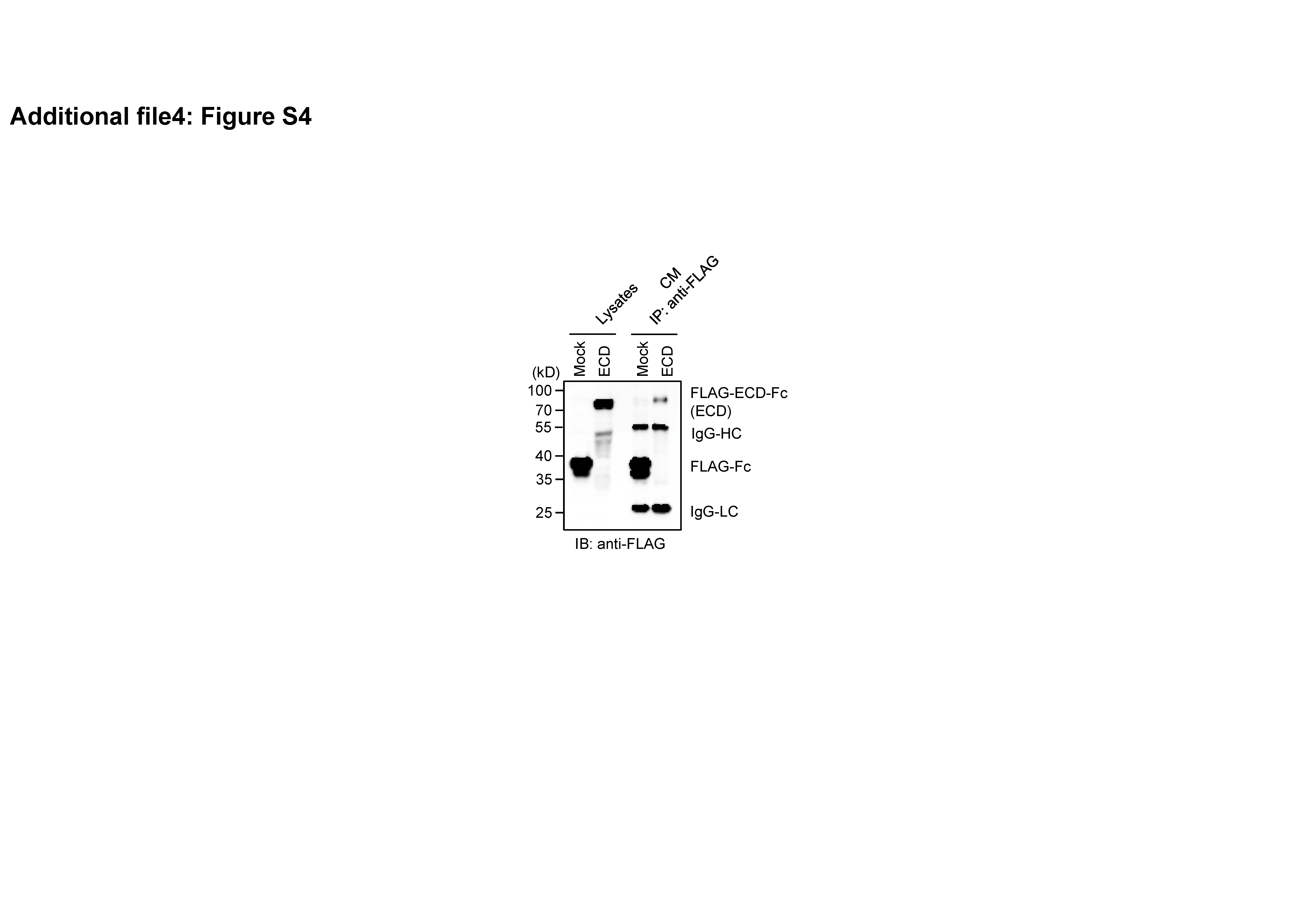

Supplement: Supplementary file 4 — Additional file 4: Figure S4. Preparation of secretable DGCR2-ECD. FLAG-hDGCR2-ECD-Fc (ECD) expression construct or empty FLAG-Fc construct (Mock) were transfected into HEK 293T cells. Cell lysates and conditional media (CM) were collected. To concentrate secreted ECD, the CM were subjected into IP with anti-FLAG antibody. IgG-HC indicates IgG heavy chain, and IgG-LC indicates IgG light chain. [file 13578_2023_1081_MOESM4_ESM.tiff]
